# Iatrogenic Pneumocephalus Resolved by Oxygen Therapy

**DOI:** 10.7759/cureus.19830

**Published:** 2021-11-23

**Authors:** Sambhawana Bhandari, Maun R Baral, Mingwei Yu

**Affiliations:** 1 Internal Medicine, Danbury Hospital, Nuvance Health, Danbury, USA

**Keywords:** cervical epidural injection and pneumocephalus, normobaric oxygen for pneumocephalus, oxygen therapy for pneumocephalus, iatrogenic pneumocephalus, pneumocephalus

## Abstract

Iatrogenic pneumocephalus and dural puncture are some causes of headache following cervical epidural injection. A 50-year-old woman presented with a sharp headache at the base of her skull following a cervical epidural injection for chronic neck pain. It was not relieved by lying down and was associated with nausea, vomiting, and photophobia without fever or neck rigidity. Neurological examination failed to show any abnormalities. A head CT scan showed newly evident pneumocephalus in the ventricular system and the extra-axial subarachnoid space within the sulci of the right frontal lobe. Oxygen supplementation was started with the help of a non-rebreather mask connected to 15 liters of oxygen and was slowly down titrated to room air. Repeat CT scan of the head after 48 hours showed complete resolution of the intracranial pneumocephalus. Normobaric oxygen therapy via a non-rebreather mask and a high-flow nasal cannula is effective for the treatment of intracranial pneumocephalus.

## Introduction

Cervical epidural injection of anesthetics and/or steroids is a commonly used procedure for cervical radiculopathy and/or pain [1]. Complications associated with this procedure are rare and usually are the result of an accidental dural puncture. This can lead to dural puncture headache, or rarely, pneumocephalus [2]. Although the exact incidence is not known, there have been only one to two cases reported per year [3]. Oxygen therapy has shown to be safe and efficacious in the treatment of pneumocephalus in terms of the rate of resorption of air and change in its volume [4]. We present a rare case of pneumocephalus following cervical epidural injection, which was successfully managed with oxygen therapy.

## Case presentation

A 50-year-old woman with a medical history of chronic neck and back pain secondary to a motor vehicle accident (MVA) in 2016, who had been receiving outpatient epidural injections for pain control, presented to the emergency room with complaints of severe headache. She reported that she had a cervical epidural injection roughly 30-45 minutes prior to this event.

She described the headache as sharp in character, constant, at the base of her skull, and 10/10 in severity with radiation to the neck and upper back. It was not relieved by lying down. Her headache was also associated with nausea, vomiting, and photophobia. She denied having a history of fever or neck rigidity.

On presentation, her vital signs were as follows: systolic blood pressure (SBP): 190 mmHg; diastolic blood pressure (DBP): 97 mmHg; pulse rate: 62 beats/min; respiratory rate: 18/min; and temperature: 37.8° Celsius (100° Fahrenheit). On examination, the patient was alert, oriented to time, place, and person, and was able to follow commands. Cranial nerves and visual field examination was negative for any focal deficits; extra-ocular muscles were intact without nystagmus; pupils were symmetrical, round, and reactive to light; the facial sensation was intact to light touch, and facial movements were full and symmetric with no dysarthria; the soft palate was elevated symmetrically; shoulder shrug strength was full bilaterally; and tongue protrusion in the midline with full side-to-side movement. On motor examination, the tone was normal in passive range of motion in the four limbs, power was normal and equal bilaterally, deep tendon reflexes were symmetric bilaterally, Babinski’s sign was negative with toes going downward bilaterally, no dysmetria in finger-nose testing, and she was able to stand without assistance. Sensory examination showed intact sensation to light touch in the bilateral upper and lower extremities.

Blood work showed the following findings: sodium: 136 mEq/L; potassium: 4.1 mEq/L; blood urea nitrogen (BUN)/creatinine: 10/0.66; glucose: 182 mg/dL; white blood cell (WBC) count: 21.2/L; hemoglobin: 14.5 g/dL; and platelets: 289/L.

**Figure 1:**
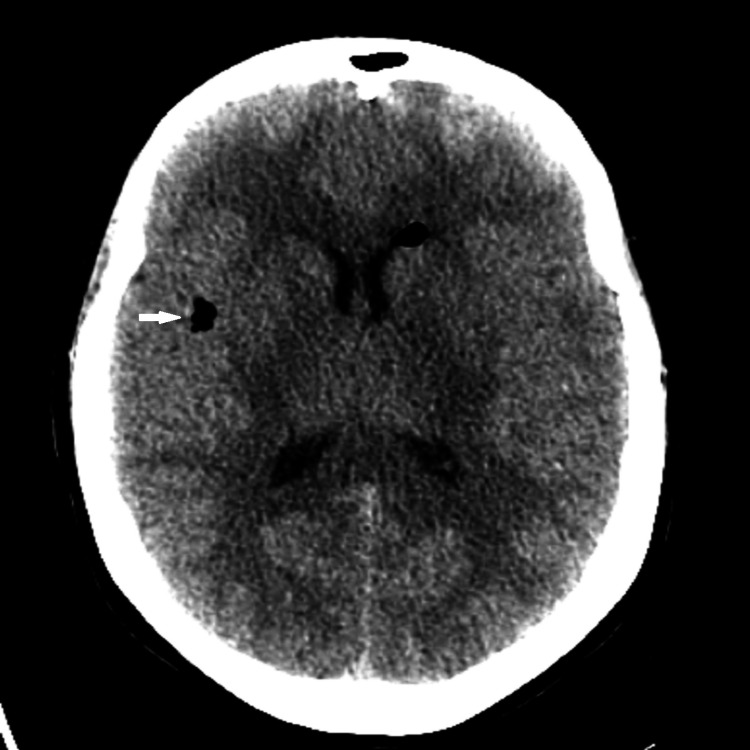
Pneumocephalus in the right frontal lobe sulcus.

**Figure 2:**
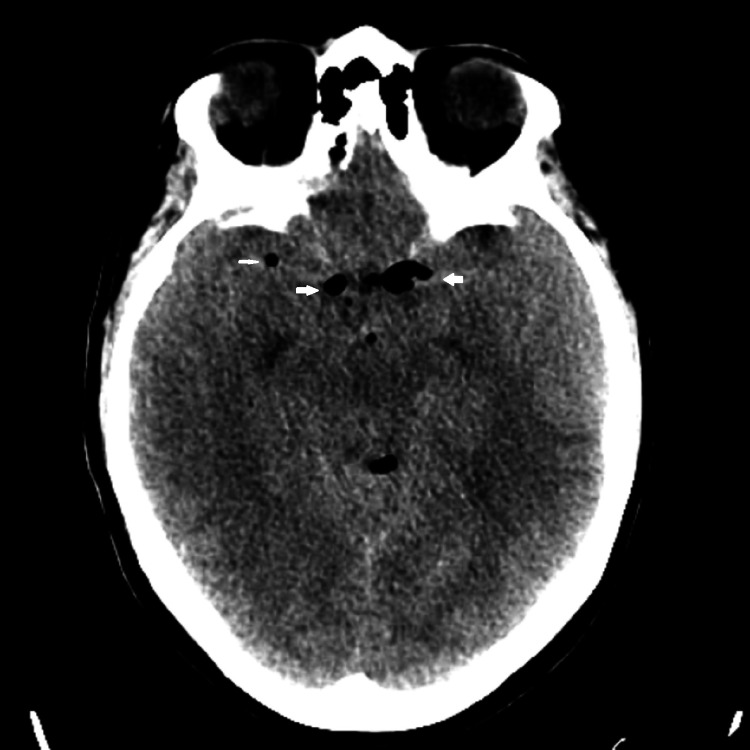
Pneumocephalus in the ventricular system.

A head computed tomography (CT) scan was performed, which showed newly evident pneumocephalus in the ventricular system and the extra-axial subarachnoid space within the sulci of the right frontal lobe (Figures 1, 2).

The patient was then admitted with a working diagnosis of pneumocephalus most likely in the setting of traumatic cervical epidural injection.

Pain control was done with butalbital (50 mg)/acetaminophen (325 mg)/caffeine (40 mg) oral tablet, with Dilaudid and ketorolac as needed. Oxygen supplementation was done with the help of a non-rebreather mask connected to 15 liters of oxygen, which was gradually tapered down to 6 liters via a high-flow nasal cannula after 24 hours.

Cervical and thoracic spine magnetic resonance imaging was performed with and without contrast to rule out epidural abscesses that showed multiple levels of spondylosis mainly prevalent in C4-C7 without evidence of epidural fluid collection.

**Figure 3:**
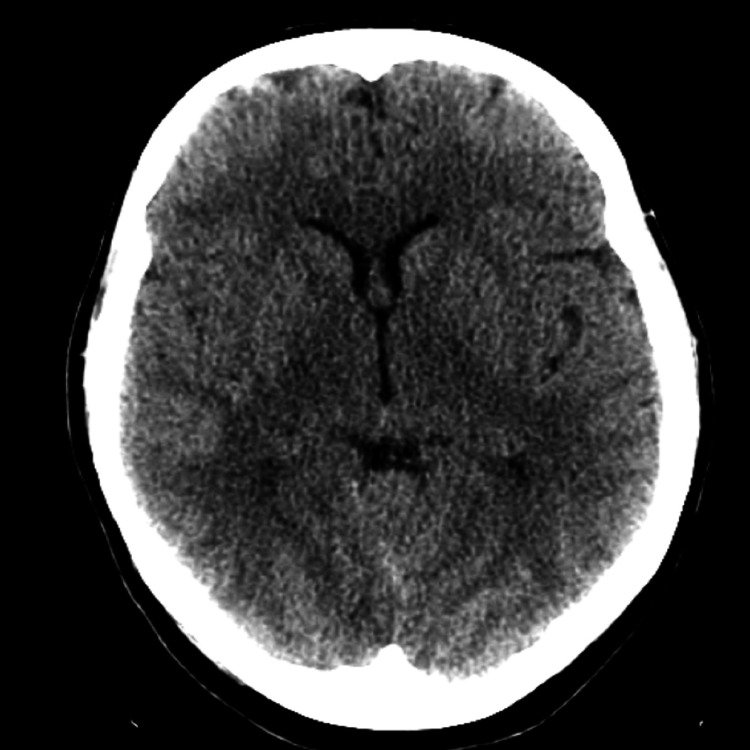
Repeat head CT scan showing resolved pneumocephalus.

A repeat CT scan of the head was done after 48 hours, which showed complete resolution of the intracranial pneumocephalus (Figure 3).

Her headache gradually improved and oxygen supplementation was then discontinued.

## Discussion

Pneumocephalus is a condition characterized by the presence of air within the cranial cavity. It can be caused by neurosurgical procedures, ENT surgeries, trauma, CNS infections by gas-producing organisms, lumbar puncture, and spinal anesthesia, and can also occur spontaneously [5].

Patients with pneumocephalus can have a wide presentation ranging from headache, motor weakness, seizures, and focal neurologic deficits depending on the distribution and amount of intracranial air [6]. The severity of the clinical presentation depends on the volume of pneumocephalus.

Post-dural puncture headache and pneumocephalus are known causes of headache after cervical epidural injection. The onset of headache is almost immediate with pneumocephalus; however, the onset with a dural puncture is typically one to three days after the procedure [2]. Pneumocephalus is similar to post-dural puncture headache in the sense that it is aggravated by any motion. However, it is not relieved by lying down, as is characteristic of post-dural puncture headache [7].

Conservative treatment with 100% oxygen and ventilator support or surgical intervention with external ventricular drain has been reported as a treatment [6]. The intracranial gas bubble has the same gas components as atmospheric and alveolar air (~79% nitrogen [N2] and ~21% oxygen [O2]). Nitrogen has a larger volume of these two gases. By oxygen supplementation, we can decrease the pulmonary nitrogen levels, thereby creating a gradient (in the blood and intracranial gas bubble), which helps the nitrogen in the pneumocephalus to diffuse into the lungs via the blood [8].

Administration of normobaric hyperoxia with a fraction of inspired oxygen (FiO2) of 100% via an endotracheal tube for three hours has shown to be safe and effective in the treatment of pneumocephalus after posterior fossa surgery in the semi-sitting position as seen in randomized control trial with 44 patients enrolled after postoperative supratentorial pneumocephalus. This study showed that with this therapy, the mean change in pneumocephalus volume and the rate of air resorption was greater in patients in the treatment arm compared to the control arm (room air) [4].

## Conclusions

Iatrogenic pneumocephalus is a rare complication of otherwise well-tolerated cervical epidural injection. It is also a complication encountered with cranial trauma and surgeries. It has a wide range of clinical presentations depending on the volume and distribution of intracranial air. Supplemental oxygen increases the rate of resorption of pneumocephalus. Normobaric oxygen therapy via a non-rebreather mask and a high-flow nasal cannula is effective for its treatment. Serial imaging is important for monitoring the amount of intracranial air.
